# Suppression of dislocations by Sb spray in the vicinity of InAs/GaAs quantum dots

**DOI:** 10.1186/1556-276X-9-278

**Published:** 2014-05-30

**Authors:** Liping Dai, Stephen P Bremner, Shenwei Tan, Shuya Wang, Guojun Zhang, Zongwen Liu

**Affiliations:** 1 State Key Laboratory of Electronic Thin Films and Integrated Devices, University of Electronic Science and Technology of China, Chengdu 610054, China; 2 Australian Centre for Microscopy and Microanalysis, The University of Sydney, Sydney 2006, Australia; 3 School of Photovoltaic and Renewable Energy Engineering, University of New South Wales, Sydney 2052, Australia

**Keywords:** Quantum dots, InAs, HRTEM, Dislocations

## Abstract

The effect of Sb spray prior to the capping of a GaAs layer on the structure and properties of InAs/GaAs quantum dots (QDs) grown by molecular beam epitaxy (MBE) is studied by cross-sectional high-resolution transmission electron microscopy (HRTEM). Compared to the typical GaAs-capped InAs/GaAs QDs, Sb-sprayed QDs display a more uniform lens shape with a thickness of about 3 ~ 4 nm rather than the pyramidal shape of the non-Sb-sprayed QDs. Particularly, the dislocations were observed to be passivated in the InAs/GaAs interface region and even be suppressed to a large extent. There are almost no extended dislocations in the immediate vicinity of the QDs. This result is most likely related to the formation of graded GaAsSb immediately adjacent to the InAs QDs that provides strain relief for the dot/capping layer lattice mismatch.

**PACS:**

81.05.Ea; 81.07.-b; 81.07.Ta

## Background

Semiconductor quantum dots (QDs) have a great potential for applications in a wide variety of novel devices [1-4]. Their optoelectronic properties can be turned by careful design through the control of their size, shape, composition, and strain [5,6]. In recent years, the III-V QDs, especially InAs/GaAs(Sb), have been drawing great interest due to their promise in wide applications beyond photovoltaics [7], such as quantum dot lasers [8,9] and photodetectors [10-12]. In particular, much effort has been dedicated to develop QD laser diodes emitting at the telecommunication bands of 1.3 and 1.55 μm. A recent promising approach is to extend the emission wavelength of self-assembled InAs/GaAs to these two regions by using a GaAs capping layer by Sb incorporation [13-16], and even a longer wavelength has already been obtained [15,16]. The strong redshift has been attributed to a type II band alignment for high Sb contents [17]. A few studies aiming to analyze the emission evolution with the amount of Sb [18,19], as well as the microstructures of these QDs, have been carried out recently by means of scanning transmission electron microscopy (STEM), atomic force microscopy (AFM), and conventional transmission electron microscopy (CTEM). The results demonstrate that they have the significant difference from those of GaAs-capped QDs [17,19-21].

However, there is almost no report about the effect of Sb sprayed on the surface of InAs immediately prior to depositing the GaAs capping layer, from the perspective of crystal structure. Since Sb incorporation will result in the formation of GaSb with a larger lattice constant, this should help provide a strain relief layer effectively bridging the lattice mismatch between InAs QDs and GaAs matrix. Then, the strain induced in the QDs during capping should be reduced, which will influence the QD size, shape, composition, defect, and dislocations. It is known that the properties of promising devices relying on quantum dot properties are compromised due to the presence of defects generated when the quantum dots are capped [22-25]. Therefore, a fundamental understanding about the defects of the QDs with and without Sb incorporation before GaAs capping is very important for device applications and will lead to better methods for minimizing the impact of these defects and dislocations. High-resolution transmission electronic microscope (HRTEM) structural imaging enables us to see atoms at their real locations and thus gives us detailed information about lattice misfit, defects, and dislocations. In this work, we used cross-sectional HRTEM to see how defects and dislocations are generated during the growth of InAs/GaAs QDs and the impact of the addition of Sb atoms.

## Methods

The two samples studied were grown by molecular beam epitaxy in an AppliedEpi GenIII system (Veeco, Plainview, NY, USA) on (100) GaAs substrates with a 200-nm-thick GaAs buffer layer. One sample with InAs/GaAs QDs capped by GaAs was named sample 1, and the other sample with InAs/GaAs QDs spayed by Sb flux for 30 s before the GaAs capping layer was named sample 2. Gallium and indium fluxes were supplied by conventional thermal sources, while As and Sb fluxes were provided by valved cracker sources. The growth rates determined by monitoring the RHEED oscillations were 0.4 and 0.035 monolayers/s for GaAs and InAs, respectively, and the measured beam equivalent pressure for Sb was 9.7 × 10^-8^ Torr. The As overpressure for all the GaAs and InAs growth steps was 2 × 10^-6^ Torr. The GaAs buffer layers of the two samples were grown at 580°C, followed by a 10-s rest, and the temperature was reduced to 500°C, and then approximately 2.0 monolayers of InAs were deposited. Different growth processes were then employed for the two samples. Sample 1 had a 30-s rest under As flow, while sample 2 was exposed to the Sb flow for 30 s. At the end of each group's spray regime, a 70-nm GaAs cap layer was grown immediately.

The structural characteristics of InAs/GaAs QDs with Sb and without Sb spray were investigated by cross-sectional HRTEM using a JEOL-JEM-3000 F microscope (Akishima-shi, Japan) operated at 300 kV. Cross-sectional TEM specimens were prepared using the standard procedures (mechanical thinning and ion milling). Fast Fourier transformation (FFT) was carried out using a DigitalMicrograph software package.

## Results and discussion

**Figure 1:**
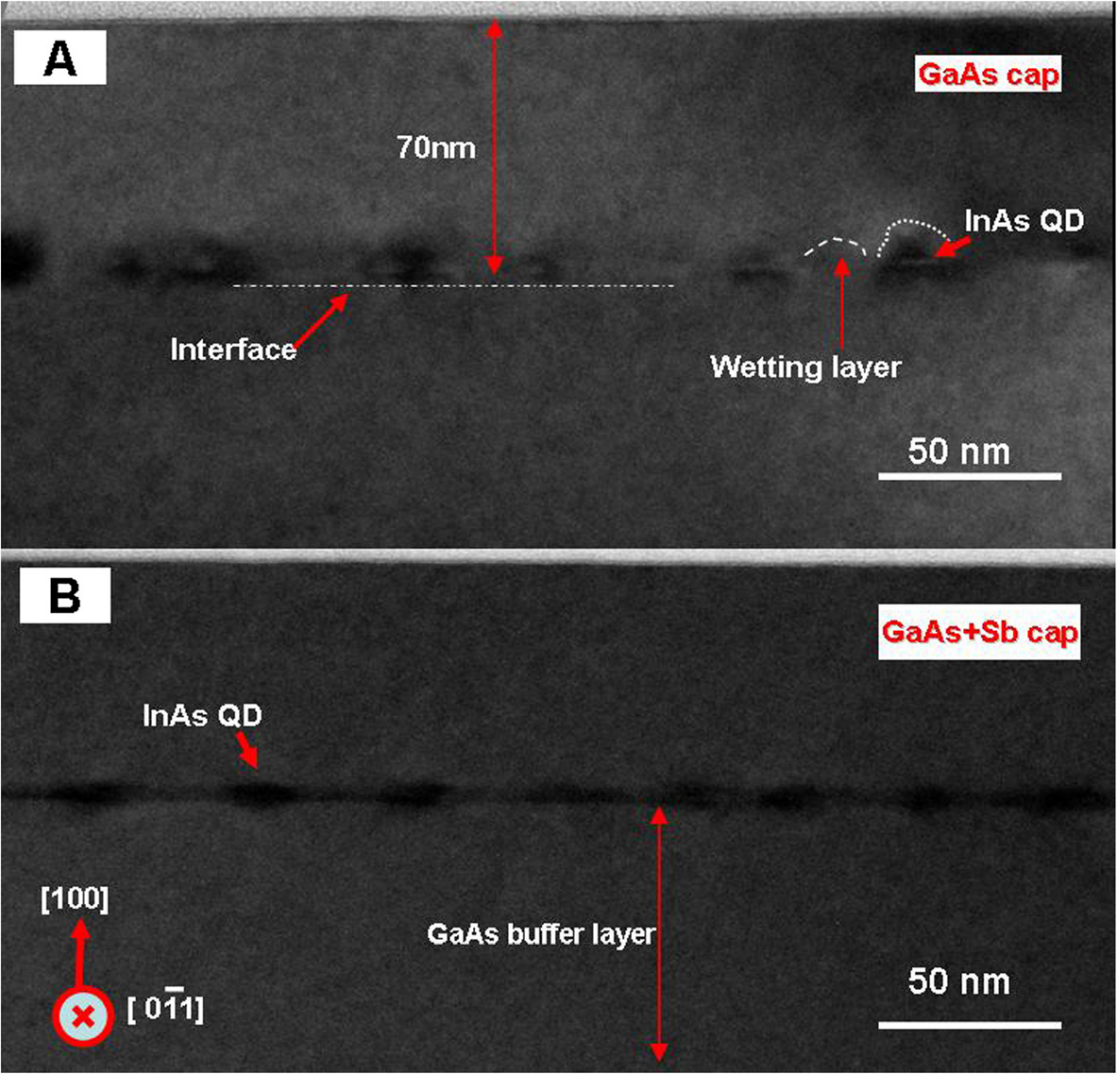
**Cross-sectional TEM images. (A)** Sample 1: InAs/GaAs QDs capped by GaAs. **(B)** Sample 2: InAs/GaAs QDs with Sb spray before the GaAs capping layer.

In order to obtain the information of the effect of Sb spray on the size, shape, and distribution of the InAs/GaAs QDs, low-magnification [1-10] cross-sectional TEM images were taken for both samples as shown in Figure 1. Sample 1 is the InAs/GaAs QD system capped by a GaAs thin film without Sb spray, and sample 2 is the InAs/GaAs QD system with Sb spray prior to the growing of the GaAs capping layer. The layer of the capped QDs can be seen in both images which appeared as dark contrast caused by the strain field around the capped InAs/GaAs QDs [25]. Clear differences in size, shape, and distribution can be seen from the two layers of InAs/GaAs QDs. The former QDs present a typical InAs QD shape close to pyramidal [26], with a height of 5 ± 1 nm and a base width of 12 ± 2 nm, and the interspacing of QDs is in the range of 15 to 25 nm. It is obvious that the Sb spray has significantly increased the density of the dots and reduced the typical QD height approximately by half. Also, the corresponding QDs show a lens shape with almost the same base width. In addition, a uniform size distribution and low coalescence frequency were also observed, with a relatively uniform areal number density of dots, consistent with results from the atomic force microscopy (AFM) analysis which showed that the areal density number density of the QDs was approximately doubled due to the Sb spray [19]. Here, the Sb changing the QD morphology is considered to be the Sb that acts as a surfactant on the growth surface as the In adatoms migrate around to form dots. Since the interface energy is decreased, InAs does not bead up as much so we get flatter QDs and we get a higher areal density. But the currently observed decrease in the height of the QDs is not consistent with other results which showed that with the Sb incorporation in the capping layer, the height of the QDs was more than twice that of the typical only-GaAs-capped QDs [20]. We believe that it is reasonable that an increase in QD density would inevitably result in a concomitant decrease in QD size with a constant of 2.0 ML of deposited InAs, similar to that using a GaAsSb buffer layer for the formation of small, uniform, and dense InAs quantum dots [27]. Here, the observed evenly distributed and uniform QDs can be attributed to the incorporation of Sb which decreased the interface mismatch between the GaAs buffer layer and InAs and hence decreased the balance strain field. The results of increase in density and the decrease in QD height imply that the addition of Sb acted as a surfactant and therefore improved the InAs QD nucleation rate and reduced the surface energy [27]. In order to determine how the addition of Sb can influence defects and dislocations, further HRTEM of the QDs was performed.

**Figure 2:**
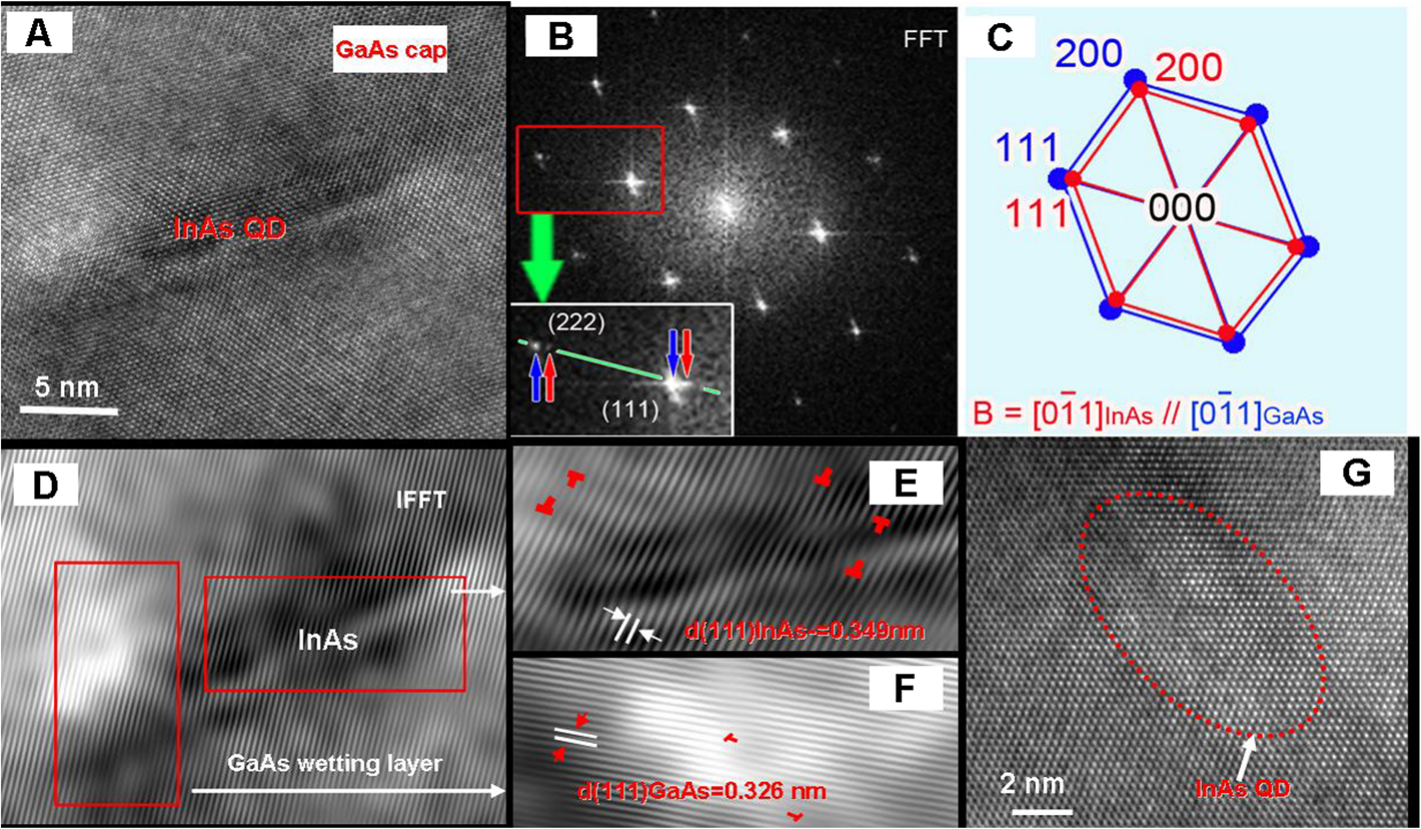
**HRTEM images of the InAs/GaAs QD with the GaAs thin film capping layer. (A)** HRTEM image showing a single QD of InAs buried in the GaAs buffer layer. **(B)** Fast flourier transformation (FFT) image of **(A)** providing electron diffractions of both GaAs and InAs phases. **(C)** Indexing of the FFT image indicating a typical molecular beam epitaxy orientation (cubic parallel orientation) between InAs and GaAs viewed at the direction $\left[0\bar{1}1\right]$. **(D)** An inverse FFT (IFFT) image formed by (111) diffraction spots. **(E)** IFFT image of InAs QD exhibits planar mismatch and dislocations marked by T symbol. **(F)** IFFT image of GaAs wetting layer exhibits lattice deformation and dislocations marked by T symbol. **(G)** HRTEM image of one small-sized QD without any dislocations.

To understand the effect of Sb spray on the structure of the InAs QDs, a number of QDs from both samples were analyzed to gain information on the size and shape of the QDs and the dislocation distribution around them. High-resolution TEM imaging was performed from two cross-sectional specimens. Figure 2A shows a typical [1-10] high-resolution TEM image of one buried InAs QD in sample 1 without Sb spay. It shows that the QD has a base width of about 13 nm and a height of about 5 nm, with dark contrast caused by the strain field around the InAs QD observed. The FFT corresponding to Figure 2A is presented in Figure 2B. The split of each diffraction spot, as shown by the inset on the lower left of Figure 2B, indicates the coexistence of GaAs and InAs phases with their crystal planes parallel to each other as schematically shown in Figure 2C.The small-scale lattice mismatch exists because of the difference in the (111) plane spacings of InAs and GaAs, as determined from the inverse FFT image (Figure 2D) formed by the (111) diffraction spots, which are 0.349 and 0.326 nm, respectively. Hence, during the epitaxial growth, the strain field would inevitably accumulate. In this case, the value of the stress would depend on the size of the QDs: the larger the size of the InAs QDs, the greater the stress accumulation. At a critical size, the accumulated stress would be relaxed, resulting in the formation of lattice deformations and/or dislocations as shown by the IFFT (111) fringes of the InAs QDs and the GaAs wetting layer (Figure 2E,F); here, the GaAs wetting layer, not to be confused with the InAs wetting layer, is the vicinity GaAs layer around QDs. The dislocations marked by the T symbols were found to be located not only at the interface and inside the InAs QDs but also in the GaAs wetting layer. A number of other InAs QDs were further analyzed. It was found that the density and distribution of the dislocations are associated to the base width and the shape of the InAs QDs. Those QDs, with a small size and a uniform shape, had less stress accumulated, and consequently, less deformation and dislocations were formed. Some of the small QDs even had no dislocations, as seen in Figure 2G.

**Figure 3:**
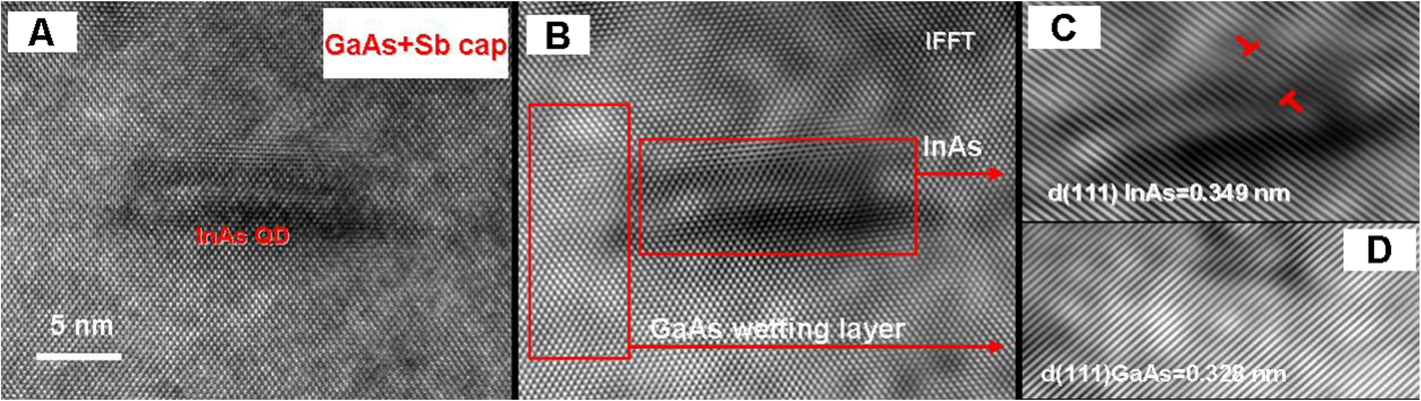
**HRTEM images of a Sb-sprayed InAs/GaAs QD with the GaAs capping layer. (A)** HRTEM image showing a single Sb-sprayed InAs QD with the GaAs buffer layer. **(B)** An IFFT image of **(A)**. **(C)** IFFT image of InAs QD exhibits (111) planar mismatch and dislocations marked by the T symbols. **(D)** IFFT image showing the GaAs (111) planes of the wetting layer without any dislocation.

In order to access the effect of the Sb spray on the defect structure of the QDs, an InAs QD of similar size and shape from sample 2 was analyzed. Its high-resolution TEM image as shown in Figure 3A shows that the QD has a base width of about 13 nm and a height of about 4 nm. A relative uniform stress field appeared around the Sb-sprayed QD, and especially, there is almost no light and dark contrast caused by the strain field in the GaAs wetting layer, indicating that less stress and dislocations were generated. These observed features are well in agreement with the IFFT analysis presented in Figure 3. Figure 3B shows the IFFT image of the QD showing undetectable lattice deformation at the interface of InAs and GaAs. An IFFT image formed by only including the (111) plane reflections revealed only two dislocations located at the interfacial region of the QD and GaAs (Figure 3C). A similar IFFT analysis was unable to detect any dislocation in the wetting layer. In other words, the addition of Sb appeared to passivate the defects in the vicinity of the QDs. This is unlike the other InAs/GaAs QD systems where defects of dislocation loops and stack faults were even observed to have penetrated the spacer layer and extended to the surface [21,28]. Our HRTEM results show that the 30-s Sb spray process that we adopted in our fabrication can greatly reduce the structural defects and dislocations of our InAs/GaAs system and prevent the formation of extended defects. The reduction of defects is undoubtedly related to the Sb incorporation in the lattice and the formation of GaSb [29]. The formation and intermixing of GaAsSb with InAs would result in less stress since the lattice misfit between InAs and GaAsSb is smaller than that between GaAs and InAs. It is known that the key impediment to the application of QD-based devices is that a good proportion of the QDs may not be active because of the non-radiative recombination through defects and dislocations around the QD-cap interface [29]. Thus, the Sb spray is expected to improve the performance of QD-based devices through minimizing the defects and dislocations in the InAs/GaAs QD system and therefore to keep many quantum dots active [30].

There have been reports of InAs and GaSb intermixing with the formation of an In_*x*_Ga_1 - *x*_ As_*y*_Sb_1 - *y*_ alloy in the core of the QDs [31]; however, it was also demonstrated that the Sb atoms are distributed solely in the As atom matrix of the QDs [20]. While the HRTEM structural imaging can allow us to see atoms at their real locations, and give us detailed information about lattice misfit, defects, and dislocations, we are exploring the feasibility of by atom probe tomography (APT) to identify how the Sb atoms distribute and interact with other atoms in and around the QDs in order to determine the exact mechanism by which the defect passivation observed in our results are realized.

## Conclusions

HRTEM has been used to study the structural properties of self-assembled InAs/GaAs QDs with and without an Sb spray immediately prior to GaAs capping. The Sb spray process can reduce the height of the InAs/GaAs QDs and increase the QD density and, more importantly, can passivate the defects and dislocations in the dot/cap interface region and suppress dislocations to a large extent. This result is very useful for fabricating novel QD-based optoelectronic devices, in particular photovoltaic devices where ensuring a high proportion of QDs that are active is a key requirement for novel energy conversion mechanisms and to reduce losses due to recombination via defects.

## Competing interests

The authors declare that they have no competing interests.

## Authors' contributions

LPD carried out the TEM experiment and analysis and drafted the manuscript. ZWL and SPB provided the design and guidance for the study and helped revise the manuscript. SWT, SYW, and GJZ provided help for the experimental preparation. All authors read and approved the final manuscript.
